# Psychiatric brain gain in Switzerland. Competency-based onboarding.

**DOI:** 10.1192/j.eurpsy.2024.807

**Published:** 2024-08-27

**Authors:** D. Gurrea Salas, R. F. Palma Álvarez

**Affiliations:** ^1^Department of Addictive Disorders, Psychiatric Services Aargau, Brugg, Switzerland; ^2^Department of Psychiatry, Hospital Universitari Vall d’Hebron, Barcelona, Spain

## Abstract

**Introduction:**

In the last 30 years, Switzerland has been established as a destination country for psychiatric trainees. The needed competences for the work as a trainee deviate regarding colleagues from foreign countries though, hindering a viable solid development professional without specific on-boarding program. A similar approach to the figure of tutor anchored in the Spanish postgraduate medical training is still missing in the Swiss medical System. Hereby we performed a survey in the new colleagues who are part from the medical team in an observer status before beginning with the responsibilities as a trainee.

**Objectives:**

Recognizing competences and needs of the onboarding in current trainees that are still allocating because of the work conditions as stated in the following paper, (Bischof et al. Swiss Arch Neurol Psychiatr Psychother. 2021;172:w03198)

**Methods:**

Survey with open questions collecting needs and competences expected to fulfil in Switzerland were distributed in 5 different medical colleagues in an observer status between August 2022 and September 2023.

**Results:**

Response rate was 62,5 %. Main reasons for the migration were considering better perspectives in education and professional development in the goal country, coming push factors as the current work situation in the original country to the fore. Support regarding the local language and an overview of the interprofessional communication were outlined as the advantage of the internship prior to the duties as a psychiatric trainee.

**Conclusions:**

An structured on-boarding program is a demand for the newcomers - majority of trainees from foreign countries - to step in better in the Swiss health system. Elements of the Spanish trainee system could be adapted for a suitable allocation and integration process in the goal country.

**Disclosure of Interest:**

None Declared

